# Glutathione S-transferases (GSTT1 and GSTM1) gene deletions in Tunisians: susceptibility and prognostic implications in breast carcinoma

**DOI:** 10.1038/sj.bjc.6601292

**Published:** 2003-10-14

**Authors:** A Khedhaier, S Remadi, M Corbex, S B Ahmed, N Bouaouina, S Mestiri, R Azaiez, A N Helal, L Chouchane

**Affiliations:** 1 Laboratoire d'immuno-oncologie moléculaire, Faculté de Médecine de Monastir, 5019 Monastir, Tunisia; 2 Laboratoire Cytopath, Sousse Tunisia; 3 Centre International de Recherche sur le Cancer (CIRC), Unité d'Epidémiologie Génétique du cancer, Lyon, France; 4 Service de carcinologie médicale, CHU Farhat Hached, Sousse, Tunisia; 5 Service de carcinologie radiothérapie, CHU Farhat Hached, Sousse, Tunisia; 6 Laboratoire de biologie clinique, CHU Fattouma Bourguiba Monastir, Tunisia; 7 Institut Supérieur de Biotechnologie de Monastir, Monastir, Tunisia

**Keywords:** GSTT1, GSTM1, polymorphism, breast carcinoma, susceptibility, prognosis

## Abstract

Glutathione S-transferase Theta1 and Mu1 (GSTT1 and GSTM1) are involved in the metabolism and detoxification of a wide range of potential environmental carcinogens. Conversely, they contribute to tumour cell survival by detoxification of numerous products induced by cancer therapy. The authors designed a large study to investigate the susceptibility and prognostic implications of the GSTT1 and GSTM1 gene deletions in breast carcinoma. The authors used the polymerase chain reaction to characterise the variation of the GSTT1 and GSTM1 genes in 309 unrelated Tunisian patients with breast carcinoma and 242 healthy control subjects. Associations of the clinic-pathologic parameters and the genetic markers with the rates of the breast carcinoma specific overall survival (OVS) and the disease-free survival (DFS) were assessed using univariate and multivariate analyses. A significant association was found between *gene deletion of* GSTT1 and the risk of early onset of breast carcinoma (OR=1.60, *P*=0.02). The lack of GSTT1 gene deletion was significantly associated with poor clinical response to chemotherapy (OR=2.29, *P*=0.03). This association was significantly higher in patients with axillary's lymph node-negative breast carcinoma (OR=12.60, *P*=0.005). The null-GSTT1 genotype showed a significant association with increased DFS in this selected population of patients. This association was even higher in patients carrying both null-GSTT1 and -GSTM1 genotypes. The *gene deletion of* GSTs may predict not only the early onset of breast carcinoma but also the clinical response to chemotherapy and the recurrence-free survival for patients with lymph node-negative breast carcinoma.

Breast carcinoma is the most frequent malignancy among women, representing a major health problem in many countries. Family history of breast carcinoma and reproductive history account for only 30% of cases (Henderson, 1993; Ghadirian *et al*, 1998). Epidemiological studies have suggested that environmental factors may play a major role in the development of breast carcinoma (Haris *et al*, 1992; Henderson, 1993). The potent carcinogens implicated in breast carcinogenesis are polycyclic aromatic hydro-carbons (PAHs), aromatic and heterocyclic amines present in the diet and environmental exposures (Calaf and Russo, 1993; Dunnick *et al*, 1995). These were shown to cause mammary tumours in rodents and to form adducts in human breast cells (Cavalieri *et al*, 1988; Byers, 1994; Dunnick *et al*, 1995; Pfau *et al*, 1998). Polycyclic aromatic hydro-carbons are activated by cytochrome P-4501A1(CYP1A1), and the resulting reactive intermediates are detoxified by glutatione-S-transferases (GSTs). These enzymes are expressed in normal breast tissue as well as in breast tumours (Sadreih *et al*, 1996; Lee *et al*, 1997; Stone *et al*, 1998). Individuals who are homozygous for the null-GSTM1 or null-GSTT1 genotypes lack the respective enzyme functions (Seidegaard *et al*, 1986; Pemble *et al*, 1994). The null-GSTM1 genotype appears to be common in several populations, whereas the null-GSTT1 genotype exhibits population frequencies that depend on ethnicity (Bell *et al*, 1993; Nelson *et al*, 1995; Lin *et al*, 1998). The GSTM1 and GSTT1 defects seem to be associated with increased risk of certain cancers (Rebbeck, 1997; Strange *et al*, 1998); however, conflicting data have been observed (Chen *et al*, 1996; Baily *et al*, 1998; Houston, 1999). This may be attributable to differences in study design and the analysed populations, as well as to the presence of different confounding factors. The Tunisian population is known for its relative homogeneity. We analysed the relationship between *gene deletion of* GSTM1 and GSTT1 and the susceptibility to breast carcinoma in this population. Chemotherapy and radiation therapy after surgery for breast carcinoma reduce the risk of recurrence and mortality. However, many patients are not cured with these treatments. Considerable research has been focused on tumour clinic-pathologic characteristics that may predict prognosis. Little is known about the possible underlying host factors that could play a substantial role in reduced treatment efficacy. Both chemotherapy and radiation therapy largely exert their antineoplastic effects by generating reactive oxygen products (Hellman, 1993; Weijl *et al*, 1997). As these are the proximate cause of tumour cell death in many cases, the amounts of reactive species that reach tumour cells and have either direct cytotoxic effects or trigger intracellular apoptotic pathways is likely to have initial and immediate impact on treatment efficacy. Thus, interindividual variability in enzymes that will affect reactive oxygen species (ROS) may have a significant impact on patient prognosis after treatment.

Several reports highlighted the role of GSTs enzymes in the detoxification of numerous products induced by cancer therapy (Hurst *et al*, 1998; Hayes and Melellan, 1999). GSTT1 and GSTM1 are active in the elimination of several products resulting from reactive oxidant damage to DNA and lipids, such as organic epoxides, and hydroperoxides. The reduction of these molecules by GSTs prevents further oxidant damage within cells. Individuals lacking each of these enzymes may have reduced removal of secondary organic oxidation products produced by cancer therapy and, thus, may have better prognoses. In line with this hypothesis, we investigated, in a large cohort of 309 breast carcinoma from whom complete follow-up data were collected, the potential association of the *gene deletion of* GSTM1 and GSTT1 enzymes with tumour clinic-pathologic characteristics and with the increased risk of relapse and death from breast carcinoma.

## MATERIALS AND METHODS

### Patients and controls

The gene and allele frequencies of the GSTT1 and GSTM1 genes were determined in a group of 242 control subjects and 309 patients with breast carcinoma. Controls and patients were selected from the same population living in the middle coast of Tunisia. Both the control and patients groups include unrelated subjects.

Clinical follow-up data were collected on the cohort of the 309 patients recruited from the department of Radiation Oncology and Medical Oncology of Sousse Hospital (Sousse Tunisia) between 1994 and 2002.

All patients included in this study had primary breast carcinoma, with unilateral breast tumours. The patients (304 females and five males) had a mean age of 52±24 years. The median of follow-up was 36 months (range, 1–120 months). At time of analysis, 76 patients relapsed (local or distant recurrence). Among them, 36 patients died from breast carcinoma (47.3%). A detailed description of the clinic-pathologic characteristics of this cohort has been reported elsewhere (Ben Ahmed *et al*, 2002).

Control subjects (110 females and 132 males) having a mean age of 41±14 years, were healthy blood donors having no evidence of any personal or family history of cancer (or other serious illness). Written informed consent was obtained from all subjects.

### Patients treated by chemotherapy as a primary treatment:

Among the 309 patients, 140 had chemotherapy as an anticancer primary treatment. This group includes 122 patients who had neoadjuvant chemotherapy and 18 patients who received chemotherapy as a palliative treatment. The chemotherapy induction was based in all cases on the combination of cyclophosphamide (100%), 5-fluorouracil (100%) with adriamycin (36%) or epirubicin (51%) or methotrexate (13%). For neoadjuvant treatment, patients received four or six chemotherapy cycles before surgery. The clinical response to induction chemotherapy for all cases was defined according to the following criteria: complete response when regression of the tumour was total, partial response when the reduction of tumour size was greater than 50% and poor response when the reduction of tumour size was less than 50% (tumour size was measured by the bidimensional product of the horizontal and vertical dimensions). Lumpectomy or mastectomy was performed 3 weeks after the last cycle of induction chemotherapy; postoperatively, patients resumed chemotherapy. Consolidative radiation therapy was performed in 26 cases.

### DNA extraction

Genomic DNA was extracted from peripheral blood leucocytes by a salting out procedure (Olerup and Zetterquist, 1992). Briefly, 10 ml of blood was mixed with triton lysis buffer. Leucocytes were spun down and washed with H_2_O. The pellet was incubated with proteinase K at 56°C and subsequently salted out at 4°C using a saturated NaCl solution. Precipitated proteins were removed by centrifugation. The DNA in the supernatant was precipitated with ethanol. The DNA pellet was dissolved in 400 *μ*l sterile distilled H_2_O.

### Polymorphism analysis of GSTT1 and GSTM1 genes

Polymorphic deletion of the GSTT1 and GSTM1 genes was revealed by PCR-based assays with *β*-interferon as a control gene.

In addition to the *β*-interferon primers (5′-GGCACAACAGGTAGTAGGCG-3′, and 5′-GCCACAGGAGCTTCTGACAC-3′), two sequence specific oligonucleotide primers (GSTT1 or GSTM1) were used for each PCR: for GSTT1, the 3′ primer (5′-TTCCTTACT GGTCCTCACATCTC-3′) was used in combination with the 5′ primer (5′-TCACCGGAT CATGGCCAGCA-3′). For GSTM1, the 3′ primer (5′-CTGCCCTACTTG ATTGATGGG-3′) was used with the 5′primer (5′-CTGGATTGTAGCAGATCATGC-3′). In all, 30 *μ*l of PCR reaction mixture was comprised of genomic DNA samples (100 ng), 200 *μ*mol l^−1^ dNTPs, 1.5 mM MgCl_2_, 1 × Taq polymerase buffer, 100 pmol of each primer and 0.5 U of Taq DNA polymerase (Amersham, Paris, France). The reaction conditions used with the thermal cycler (Biometra, Göttingem, Germany) were as follows: for GSTT1, the initial incubation at 94°C for 4 min, followed by 30 cycles of incubation at 94°C for 1 min; 66°C for 1 min, and 72°C for 1 min and followed by a final incubation at 72°C for 5 min. For GSTM1, the initial incubation at 94°C for 5 min, followed by 30 cycles of incubation at 94°C for 30 s; 60°C for 1 min, and 72°C for 1 min and followed by a final incubation at 72°C for 5 min.

The reaction products were separated on a 2% agarose gel and stained with ethidium bromide. The absence of amplified GSTT1 (480 bp) or GSTM1 (271 bp) product (in the presence of the interferon PCR product (170 bp) indicated the respective null genotype for each.

### Statistical analyses

The *χ*^2^ test was used to evaluate for a significant association between disease (breast carcinoma *vs* controls) and GSTT1 or GSTM1 genotypes.

Disease-free survival (DFS) was defined as the time from the date of diagnosis to the first local or distant recurrence or to last contact. Breast carcinoma-specific overall survival (OVS) was defined as the time from the date of diagnosis to death if the patient died from breast carcinoma or to last contact. Six-year survival rates were estimated, and survival curves were plotted according to Kaplan and Meier (1958). The differences between groups were calculated by the log-rank test (Peto *et al*, 1977).

Clinicopathological parameters were dichotomised as follows: nodal status (⩾1 *vs* no positive lymph node), SBR (Scarff, Bloom and Richardson) tumour grade (1–2 *vs* 3), clinical tumour size (T_1_–T_2_ *vs* T_3_–T_4_).

Statistics were performed using SEM-STATISTIQUES software (Centre Jean Perrin, Clermont–Ferrand, France).

## RESULTS

### Polymorphisms in GSTT1 and GSTM1 genes and breast carcinoma

The distribution of the GSTT1 and GSTM1 genotypes in the patient and in the control groups is shown in Table 1

**Table 1:** GSTT1 and GSTM1 genotype frequencies in control subjects and in patients with breast carcinoma

	**Controls (*n*=242)**		**All patients (*n*=309)**			**PreM (*n*=197)**			**PostM (*n*=112)**		
**Genotype**	***n***	***(f)***	***n***	***(f)***	***P***	***n***	***(f)***	***P***	***n***	**(*f*)**	***P***
*GSTT1*											
Null	63	(0.260)	103	(0.333)	0.06	71	(0.360)	**0.02**	32	(0.285)	NS
Present	179	(0.740)	206	(0.666)	126	(0.640)	80	(0.715)			

*GSTM1*											
Null	138	(0.570)	166	(0.537)	NS	110	(0.558)	NS	56	(0.500)	NS
Present	104	(0.430)	143	(0.463)	NS	87	(0.442)	56	(0.500)		

*GSTT1 and GSTM1 combined*											
Both Null GTT1-GSTM1	36	(0.149)	55	(0.178)	NS	38	(0.193)	NS	17	(0.152)	NS
GSTT1 and/or GSTM1 Present	206	(0.851)	254	(0.822)	NS	159	(0.807)	95	(0.848)		

The *χ*^2^ test was used to determine whether significant differences (*P*-value) were observed when the patient group was compared with the control group. PreM=premenopausal patients; PostM=postmenopausal patients; *f*, frequencies; NS: not significant.

. The frequency of the null-GSTT1 genotype was 0.333 in the group of patients with breast carcinoma and 0.260 in control subjects (OR=1.42, *P*=0.06). No significant differences in null-GSTM1 distributions were seen between patients and controls (OR=0.87, *P*>0.05). The frequency of the double null-GSTT1-GSTM1 was slightly higher in patients (0.178 *vs* 0.149, OR=1.24), but the difference did not reach statistical significance. When we stratified the patients according to their menopause status, we found that the null-GSTT1 genotype frequency was significantly higher in premenopausal patients than in controls (0.360 *vs* 0.260, OR=1.60, *P*=0.02). Thus, there was an association between the presence of the null-GSTT1 genotype and the early onset of breast carcinoma. None of the GSTM1 genotypes, either alone or in combination with GSTT1 genotypes, was associated with breast carcinoma in the different subgroups.

### Prognostic significance of polymorphisms in GSTT1 and GSTM1 genes

Among the 309 patients with breast carcinoma, 140 had chemotherapy as an anticancer primary treatment, including 122 patients treated by neoadjuvant chemotherapy. A total of 53 patients were axillary's lymph node-negative. Table 2

**Table 2:** Associations between GSTT1 and GSTM1 genetic deletions and clinical response to chemotherapy induction

	**Chemotherapy response**									
	**All patients (*n*=140)**					**ALN-negative (*n*=53)**				
**Genotype**	**Poor**		**Complete/partial**			**Poor**		**Complete/partial**		
	***n***	**(*f*)**	***n***	**(*f*)**	***P-*value**	***n***	**(*f*)**	***n***	**(*f*)**	***P-*value**
*GSTT1*										
Null	13	(0.217)	31	(0.388)	**0.03**	01	(0.052)	14	(0.412)	**0.005**
Present	47	(0.783)	49	(0.612)	18	(0.948)	20	(0.588)		

*GSTM1*										
Null	29	(0.483)	47	(0.588)	NS	09	(0.474)	19	(0.589)	NS
Present	31	(0.516)	33	(0.412)	10	(0.526)	15	(0.441)		

*GSTT1 and GSTM1 combined*										
Both Null GTT1-GSTM1	05	(0.083)	17	(0.212)	**0.04**	00	(0.000)	08	(0.235)	**0.02**^a^
GSTT1 and/or GSTM1 Present	55	(0.917)	63	(0.788)	19	(1.000)	26	(0.765)		

The *χ*^2^ test was used to determine whether significant differences (*P*-value) in the clinical response to chemotherapy were observed between carriers of different genotypes. NS: not significant; *f*: frequencies; ALN=axillary's lymph node-negative.

a Fisher's test was used.

shows the associations between *gene deletion of* GSTT1 and GSTM1 and the clinical response to chemotherapy induction. Poor response to chemotherapy was seen significantly more frequently in patients having the ‘present’-GSTT1 genotype compared to those carrying the null-GSTT1 genotype (OR=2.29, *P*=0.03). This association seems to be particularly high for patients who were node-negative (OR=12.60, *P*=0.005). Poor response to chemotherapy was seen slightly more frequently in patients carrying the ‘present’-GSTM1 genotype than those with the null-GSTM1 genotype, but without reaching statistical significance (0.516 *vs* 0.483, *P*=0.19).

We were interested in the combined effects that null genotype for both GSTM1 and GSTT1 may have had on the clinical response to chemotherapy. Poor response to chemotherapy was significantly less frequent in patients carrying the double-dose null-GSTT1-GSTM1 genotype than in patients without (OR=2.97, *P*=0.04). No patient who was node-negative and carrying the double null-GSTT1-GSTM1 genotypes had poor response to chemotherapy. In contrast, 19 patients, among 45 lacking the double null genotypes had poor clinical response (*P*=0.02).

Table 3

**Table 3:** Clinicopathological characteristics of the 309 breast carcinoma and the corresponding univariate analysis of death (OVS) and relapse (DFS)

		**Breast carcinoma-specific OVS**		**DFS**	
	**%**	**6-year rate**	***P*-value**	**6-year rate**	***P*-value**
*Clinical tumour size*					
T_1_–T_2_	62.28	86.76	<0.03	79.4	<0.0001
T_3_–T_4_	37.72	72.05	38.2		

*Lymph node status*					
N(−)	54.2	94.11	<0.001	76.47	<0.01
N(+)	45.8	69.12	57.35		

*SBR grading*					
1–2	61.2	87.3	< 0.01	59.7	<0.04
3	38.8	58.3	36.1		

*Age (years)*					
<50	60.26	80.88	NS	73.5	NS
⩾50	39.74	86.76	73.5		

Six-year survival rates were estimated according to Kaplan and Meier (1958). The log-rank test (Peto *et al*, 1977) was used to determine whether significant differences (*P*-value) were observed between subgroups of patients. The lymph node status was determined based on the pathological examination. NS: not significant.

shows the clinicopathological characterisation. The distribution of the clinicopathological markers was in agreement with previously reported data, indicating that our cohort (309 patients) was representative of breast carcinoma patients. Disease-free survival and breast carcinoma-specific OVS rates were estimated and compared by univariate analysis on these clinicopathological parameters. Significant associations were found for clinical tumour size, nodal status and tumour grading with DFS and OVS. No significant differences were observed for age.

When we tested the relationship between the presence of null-GSTT1 and/or null-GSTM1 genotypes in all 309 patients and the survival (OVS or DFS), no significant differences were observed between the different Kaplan–Meier survival curves (data not shown).

We conducted further analyses to explore whether the GSTs gene deletion was associated with survival on a selected population of patients. We selected patients with axillary's lymph node-negative from the total patient population. No significant differences were seen between different OVS curves, indicating lack of association between GSTs gene deletion and OVS in this selected population.

Figure 1

**Figure 1:**
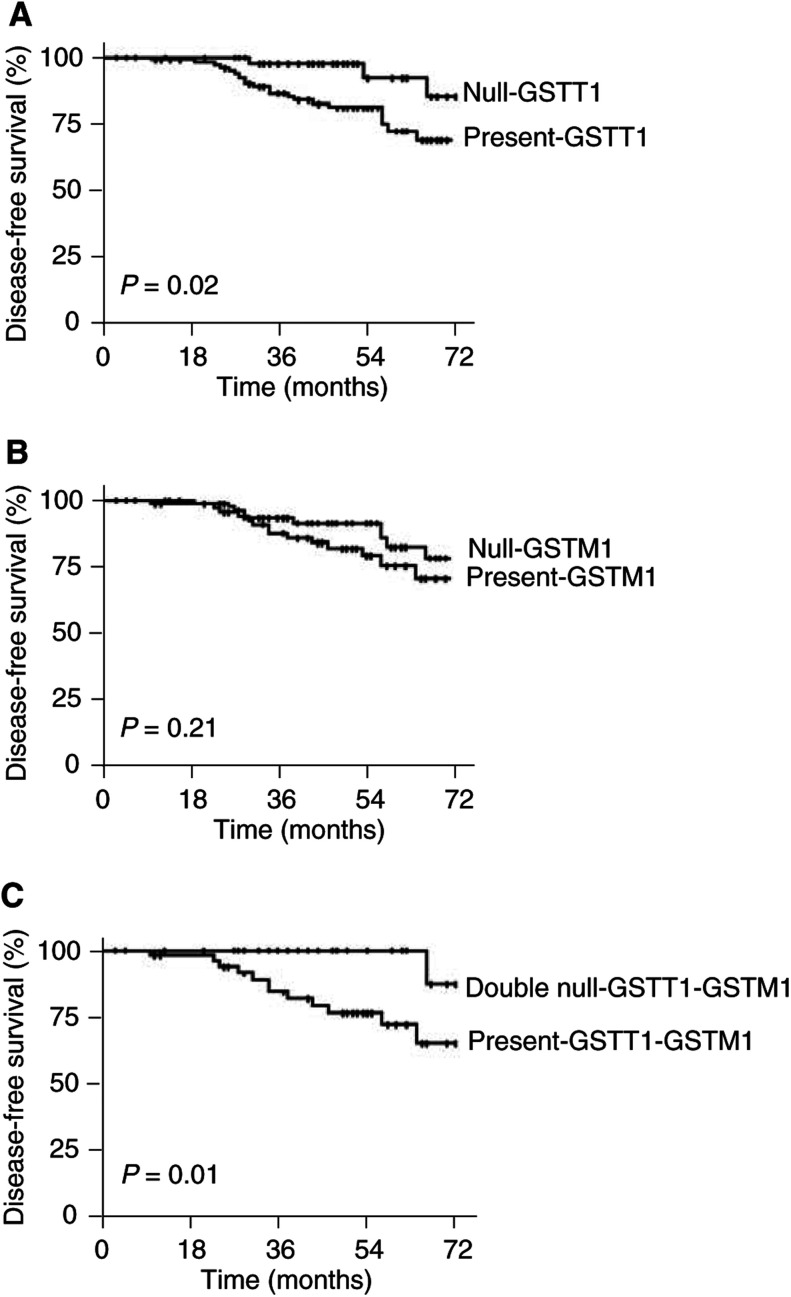
Breast carcinoma-specific DFS of axillary's lymph node-negative breast carcinoma patients according to the presence or absence of the null-GSTT1 genotype (**A**), that of null-GSTM1 genotype (**B**) and that of the double null-GSTT1-GSTM1 genotype (**C**). *P* denotes the log-rank test value.

shows the breast carcinoma-specific DFS of breast carcinoma in patients with axillary's lymph node-negative according to the presence or absence of the null-GSTT1 and/or null-GSTM1 genotypes. The DFS was significantly longer in the group of patients carrying the null-GSTT1 genotype (Figure 1A). The estimated 3- and 6-year DFS rates in the groups of patients carrying the ‘present’-GSTT1 genotype or carrying the null-GSTT1 genotype were, respectively, 85 and 61% *vs* 98 and 83.5% (log-rank test, *P*=0.02).

Although differences in DFS were seen between patients carrying the ‘present’-GSTM1 genotype and those with null-GSTM1 genotype, they did not reach statistical significance (Figure 1B, log-rank test, *P*=0.21). When DFS comparison was made between patients who had both ‘present’-GSTT1 and GSTM1 genotypes and those with both null-GSTT1-GSTM1 genotypes (Figure 1C), an increase in DFS was seen for patients carrying both null-GSTT1 and GSTM1 genotypes. The 6-year DFS rate in the group of patients with ‘present’-GSTT1-GSTM1 genotypes was 65.7 and 87.1% in that of patients with null-GSTT1-GSTM1 genotypes (log-rank test, *P*=0.01).

## DISCUSSION

Several studies have addressed the role of GSTT1 and GSTM1 gene deletions as risk factors in breast carcinoma, but the results are conflicting (Helzlsouer *et al*, 1998; Gumundsdottir *et al*, 2001; Krajinovic *et al*, 2001; Mitrunen *et al*, 2001). The ethnic heterogeneity of the analysed populations may be among the confounding factors. Hence, in this study, we assessed the potential association of these genetic polymorphisms with breast carcinoma in the Tunisian population, known for its relative homogeneity. The findings, which showed that GSTs enzymes play crucial role in the detoxification of numerous products induced by cancer therapy, prompted us to evaluate the prognostic significance of GSTs deletions in breast carcinoma.

The present case/controlled study showed a borderline significant increase in the risk of breast carcinoma in unselected subjects carrying the null-GSTT1 genotype. This association becomes clearly significant for premenopausal women. Thus the GSTT1 deletion seems to be associated specifically with the early onset of breast carcinoma. No direct correlation was found between polymorphism in the GSTM1 gene and the breast carcinoma onset in Tunisians. Our data provide evidence against a substantially increased risk of breast carcinoma associated with GSTM1 homozygous gene deletion.

The generation of ROS and their byproducts is a large part of the cytotoxic activity of chemotherapy agents. Several studies have shown that patients treated with a wide range of chemotherapy agents have marked increases in lipid peroxidation products (Subramaniam *et al*, 1993; Look and Musch, 1994; Faber *et al*, 1995; Faure *et al*, 1996). All patients of this study were treated primarily with cyclophosphamide and 5-flurouracil. There is evidence that these agents, particularly cyclophosphamide, result in lipid peroxidation and generation of ROS. The mechanism by which cyclophosphamide kills tumour cell through ROS is demonstrated by rodent data showing that the lung injury associated with treatment with cyclophosphamide is attributable to its ability to generate free radicals (Patel, 1990; Venkatesan and Chandrakasan, 1995). The GSTT1 and GSTM1 enzymes have been shown to have removal activity toward lipid hydroperoxides. The lack of these enzymes could conceivably be associated with better response to chemotherapy. In this study, we initiate the prognostic significance evaluation of the GSTs deletions by investigating the potential association between GSTT1 and GSTM1 gene deletion and the clinical response to chemotherapy induction. This evaluation indicated that only GSTT1 gene deletion is associated with the clinical response to chemotherapy. This prognostic significance was particularly high for patients with axillary's lymph node-negative breast carcinoma. Although no significant association was found between GSTM1 gene deletion and the response to chemotherapy, a combined effect of GSTT1 and GSTM1 gene deletions was seen on the response to chemotherapy induction. Indeed, none of the patients with lymph node-negative and carrying the double null-GSTT1-GSTM1 genotype had poor response to chemotherapy. In studies of haematopoietic cancers, reduced risk of disease recurrence was noted among children with acute lymphoblastic leukaemia, who had alleles encoding no or lower activity for GSTT1, -M1 and -P1 (Stanulla *et al*, 2000). An increased therapy-related toxicity among the GSTT1-null patients with acute myelocytic leukaemia has been shown (Davies *et al*, 1999). These findings support the hypothesis that patients with GSTT1-null genotypes have reduced detoxification of therapeutic agents and, in the case of high-dose therapy for acute myelocytic leukaemia, worse outcomes. In the present study of primary breast carcinoma patients, the better response to chemotherapy that was observed among GSTT1-null patients, who were not treated with high-dose therapy, can be explained by the increased efficacy of treatment.

There have been only a few studies of GST genetic polymorphism and survival after treatment of breast carcinoma (Lizard-Nacol *et al*, 1999; Ambrosone *et al*, 2001). For the most part, prior studies were undertaken on a small or heterogeneous population. In our data, the effect of GSTT1 and GSTM1 gene deletions on survival after the treatment of breast carcinoma was not evident in the entire population. The selection of axillary's lymph node-negative breast carcinoma allowed the appearance of a significant association between DFS of breast carcinoma and the GSTT1 gene deletion. No association was found with the GSTM1 gene deletion. However, there was an increase in DFS for patients carrying both gene deletions for GSTT1 and GSTM1.

In conclusion, this study suggests that GSTT1 gene deletion may be an attractive susceptibility marker for the early onset of breast carcinoma. This genetic marker represents not only a predictor of chemotherapy response but also a prognostic variable for predicting relapse in patients with lymph node-negative breast carcinoma.
